# UHF Signal Processing and Pattern Recognition of Partial Discharge in Gas-Insulated Switchgear Using Chromatic Methodology

**DOI:** 10.3390/s17010177

**Published:** 2017-01-18

**Authors:** Xiaohua Wang, Xi Li, Mingzhe Rong, Dingli Xie, Dan Ding, Zhixiang Wang

**Affiliations:** 1 State Key Laboratory of Electrical Insulation and Power Equipment, School of Electrical Engineering, Xi’an Jiaotong University, Xi’an 710049, China; xhw@mail.xjtu.edu.cn (X.W.); xiedingli@stu.xjtu.edu.cn (D.X.); dannyding@stu.xjtu.edu.cn (D.D.); Zhixiang.Wang@liverpool.ac.uk (Z.W.); 2 Department of Electrical Engineering and Electronics, University of Liverpool, Brownlow Hill, Liverpool L69 3GJ, UK

**Keywords:** gas-insulated switchgear (GIS), chromatic, partial discharge (PD), ultra-high frequency (UHF), propagation characteristics, pattern recognition

## Abstract

The ultra-high frequency (UHF) method is widely used in insulation condition assessment. However, UHF signal processing algorithms are complicated and the size of the result is large, which hinders extracting features and recognizing partial discharge (PD) patterns. This article investigated the chromatic methodology that is novel in PD detection. The principle of chromatic methodologies in color science are introduced. The chromatic processing represents UHF signals sparsely. The UHF signals obtained from PD experiments were processed using chromatic methodology and characterized by three parameters in chromatic space (*H*, *L*, and *S* representing dominant wavelength, signal strength, and saturation, respectively). The features of the UHF signals were studied hierarchically. The results showed that the chromatic parameters were consistent with conventional frequency domain parameters. The global chromatic parameters can be used to distinguish UHF signals acquired by different sensors, and they reveal the propagation properties of the UHF signal in the L-shaped gas-insulated switchgear (GIS). Finally, typical PD defect patterns had been recognized by using novel chromatic parameters in an actual GIS tank and good performance of recognition was achieved.

## 1. Introduction

In electrical power system, gas-insulated switchgears (GIS) are used extensively, and play a pivotal role in power transmission. Partial discharge (PD) in GIS lead to insulation failure, thus, the detection of PD is important in condition-based maintenance. The ultra-high frequency (UHF) method is used with high sensitivity and immunity to noise, so it is very efficient to assess the insulating status of GIS [1,2,3,4].

A substantial amount of investigation into, and results on, the UHF method have been conducted. Some of the important components of the UHF technique are the characteristics of the typical PD defects, which can be recognized and classified by a number of discriminatory attributes or features [5]. Research on the feature extraction of UHF signal has been done extensively [6]. Li presented a PD recognition system based on the *ϕ-q-N* pattern in a gray level image. The statistical features of *ϕ-q-N* images are extracted, however, the loss of some details of PD images leads to the feature errors [7]. Evagorou et al. used wavelet packet transformation and extracted the moments of the probability density function (PDF) of the wavelet coefficients at various scales as a fingerprint for PD characterization [8]. The suggested algorithm achieves encouraging results of PD classification. Hao et al. used the wavelet decomposition of phase-resolved partial discharge (PRPD) pattern. Principal component analysis is adopted to reduce the dimensionality of the data [9]. The proposed method required less than a minute to process data containing up to 8000 pulses, having 256 digital samples each. Gao et al. used the amplitude of the time-domain signal and three frequency bands in the spectrum as features of PD. The main frequency bands of the discharge are discovered, and the energy distribution is presented using the ternary plot in a novel manner [10]. Time-frequency analysis methods help to obtain the component of the specific frequency and the property of the frequency content varying over time. The energy density distribution of UHF signals on the time-frequency plane changes significantly over different UHF sensors that can represent the characteristic of the specific signal [11].

Phase-resolved data, PD gray level image, wavelet coefficients, and time-frequency distribution are used as features in these methods that achieve good effects. However, the proposed algorithms are complicated and the volume of the results is large regardless of the time domain, frequency domain, time-frequency domain, or statistical features. It is necessary to investigate the simple and efficient processing method to extract features of PD signals. In addition, some works have designed PD test cells to simulate the typical defects. The investigation of PD defect classification is significant using the actual GIS tank.

In this article, a novel methodology for processing UHF signals and revealing the features of UHF signals acquired by PD experiments in a 252 kV GIS bus-bar tank is presented, which is based on chromatic monitoring techniques [12]. The chromatic parameters are utilized to classify the three PD defect patterns using a support vector machine (SVM). Chromatic monitoring methods were extended from color science. They can be used for movement detection [13], absorption spectra analysis [14], and monitoring of semiconductor plasma processing [15]. Ragaa et al. have suggested the chromatic approach to provide PD signal discrimination based upon the phase-resolved signal amplitude [16,17]. Phase-resolved signals provide helpful feature extraction, whereas the complete wave shape processing enables detailed analysis of signal characteristics of individual single pulses [1]. The chromatic monitoring has not been used to the UHF complete pulse wave processing. Parameters such as *H* (hue), *L* (lightness), and *S* (saturation) have been used for characterization of one-dimensional signals. This reduces the amount of calculation and the size of data, meanwhile, the parameters have clear physical meanings. It acquires remarkable effect when used to process the UHF signal. In this investigation, UHF signals obtained in a PD experimental platform are processed using chromatic methodology.

## 2. Experimental Setup

**Figure 1:**
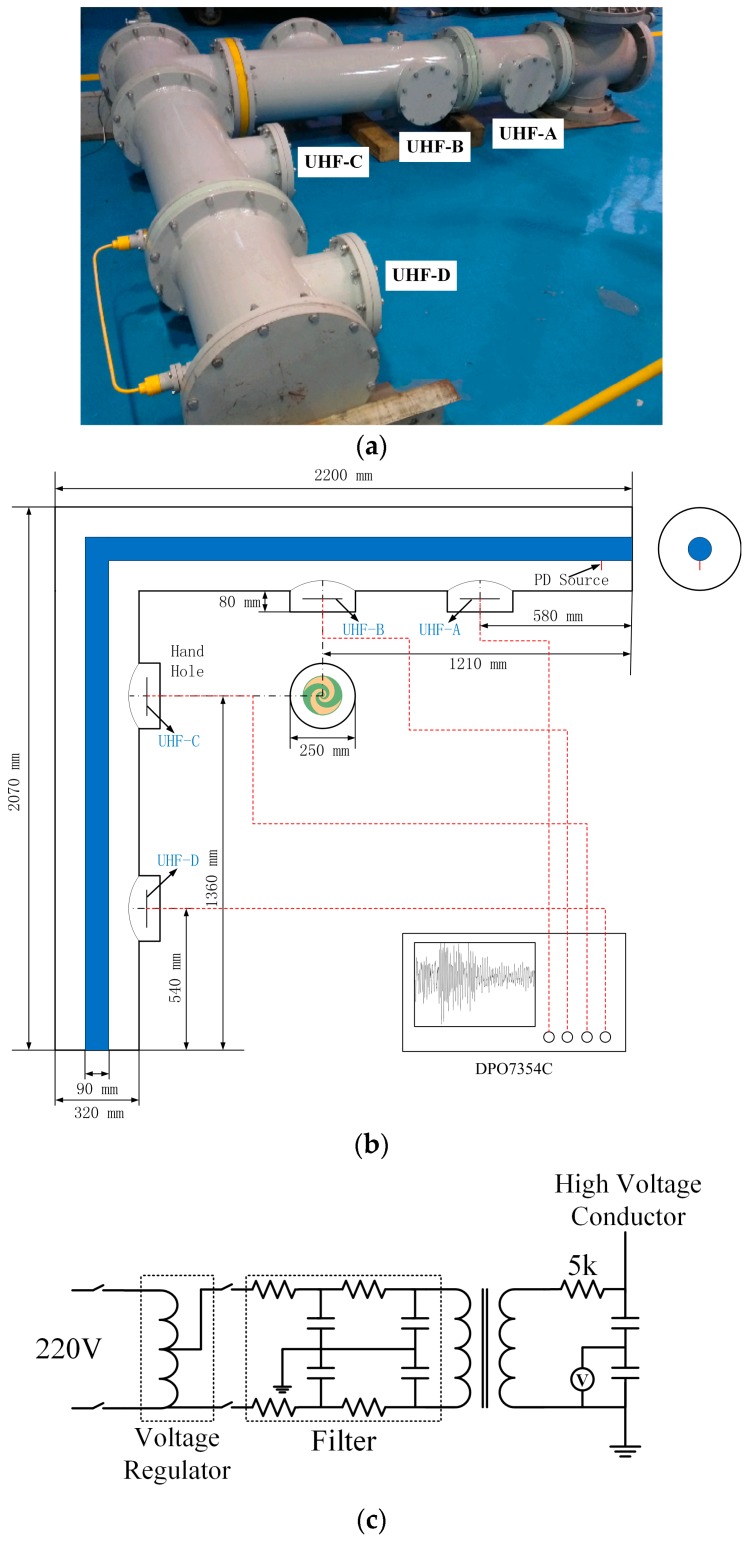
Experimental Setup: (**a**) GIS tank and placements of UHF sensors; (**b**) geometrical dimensions of GIS tank; and (**c**) schematic diagram of experimental circuit.

The experimental platform is a type of 252 kV GIS bus-bar tank with coaxial cylindrical high voltage conductor (HVC). The main structure is an L-shaped tank as Figure 1a shows. The diameters of the HVC and tank are 90 mm and 320 mm, respectively. There is an insulating bushing to introduce the applied high voltage source which is a non-PD testing transformer (Xinyuan Electric, Yangzhou, China, YDTW-30/150, capacity: 30 kVA, maximum voltage: 150 kV). The artificial PD defect is arranged in the four-way tank under the bushing. The voltage source is connected to the upper terminal of the bushing [18].

The four UHF sensors are installed inside the GIS chamber and the placements of them are shown in Figure 1a. Planar equiangular spiral antennas (PESA) with impedance transformers are utilized in this work which are studied in previous report [19]. The plane where the antennas are located is parallel to the axis of the GIS bus-bar. The four sensors are named UHF-A–UHF-D according to the distance with defect positions from the near to far. The output UHF signal is sampled by a four-channel digital oscilloscope (Tektronix, Beaverton, USA, DPO7354C, 3.5 GHz, 40 GS/s). The geometrical dimensions of the GIS tank in millimeters are illustrated in Figure 1b where the insulator spacers are omitted. The schematic diagram of experimental circuit is shown in Figure 1c.

The floating electrode defect is a kind of common defect that can lead to PD in GIS. In this section, two adjacent copper nuts are fixed on an insulated bolt, which is mounted on the central conductor as the floating electrode defect [20]. The relative angle position of the defect is 0 degrees to the antenna. The partial discharge UHF signals are recorded when the pressure of the SF_6_ is 0.1 MPa.

**Figure 2:**
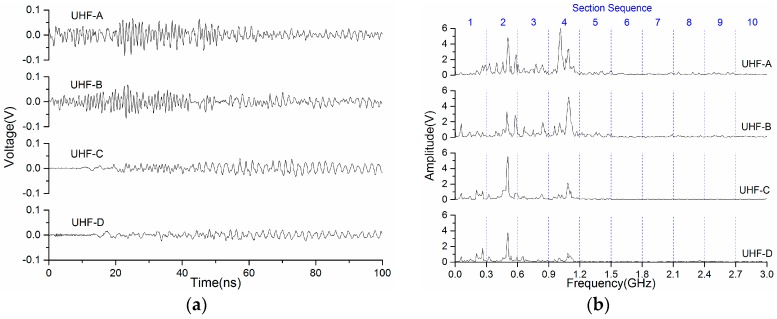
UHF signals acquired by four sensors: (**a**) time domain; and (**b**) frequency domain.

Figure 2a,b indicate waveforms of the UHF signals that are acquired by the four sensors UHF-A to D, and the results of Fourier transform. The range of the frequency spectrum is from 0 to 3 GHz. Due to the cutoff frequencies of the propagation modes of the electromagnetic (EM) wave, there are peaks in the frequency spectrum. It can be seen that the TE_11_ and TE_21_ mode are the predominant components whose cutoff frequencies are 0.47 GHz and 0.93 GHz, respectively.

The highest peaks of the signals of UHF-A and UHF-B in the frequency spectrum are in the proximity of 1.1 GHz. For UHF-C and UHF-D, the high-frequency components attenuate after passing the L branch. The peaks which are in proximity to 1.1 GHz reduce, obviously; nevertheless, the component near 500 MHz has increased and is larger than the component near 1.1 GHz.

The range of UHF is from 300 MHz to 3 GHz, the spectra are divided into 10 sections with a range of 0.3 GHz, as Figure 2b shows. More points are helpful to illustrate the similarity and correlation between standard and chromatic parameters. Furthermore, the electromagnetic wave mode is in the specific frequency band [21]. The parameters of the important sections, such as Sections 2 and 4, characterize the TE_11_ and TE_21_ modes. It is helpful to investigate the propagation characteristics of the PD-inspired EM wave in GIS. In Figure 2b, most of the energy of the signal distributes at Sections 1–5. The peaks in the frequency spectrum mentioned above are located at sections corresponding the frequency bands.

## 3. Chromatic Methodology

### 3.1. Signal Principal Parameters

The goal of UHF signal processing is to attempt to recognize signals f(t) (discrete form: f[n]) in terms of some signal characteristics. A simple way to characterize a signal is to consider its mean localizations and dispersions in the frequency domain. For this purpose, the parameters are applied in practice [12,22]; the signal energy content is expressed as:

(1) $$E_{f}=\sum_{n=1}^{N}{\left|f\left[n\right]\right|}^{2}\Delta t=\frac{1}{2\pi }\sum_{m=1}^{M}{\left|F\left[m\right]\right|}^{2}\Delta \omega$$

where F[m] is the sequence of Fourier transform of f[n], t is the time, and ω is the frequency:

(2) $$\omega_{c}=\frac{\sum_{m=1}^{M}\omega \left[m\right]{\left|F\left[m\right]\right|}^{2}\Delta \omega }{E_{f}}$$

is the average frequency of f[n]; and (RMS bandwidth)^2^:

(3) $$B^{2}=\frac{1}{E_{f}}\sum_{m=1}^{M}{\left(\omega \left[m\right]-\omega_{c}\right)}^{2}{\left|F\left[m\right]\right|}^{2}\Delta \omega$$

The parameters defined by Equations (1)–(3) provide features of signal recognition that can be considered the characteristic values of UHF signals.

### 3.2. Color and Chromatic Space

Color is the visual sensory property corresponding in humans to the categories called *red*, *green*, *blue*, etc. Color derives from the spectrum of light which means both distribution of light power and wavelength interacting in the eye with the spectral sensitivities of the light receptors. For example, the wavelength, frequency and energy of light of the color *red* are 700 nm, 428 THz, and 1.77 eV, respectively [23].

Chromatic monitoring makes an analogy with the signal and color. The parameters of the signal processed by chromatic method are related to the color features. HLS is a cylindrical-coordinate color model which rearranges the geometry of RGB and tries to be more intuitive and perceptually relevant than the Cartesian representation. It is used in many aspects such as color pickers, image editing, image analysis, and computer vision. In color science it is the convention to constitute the chromatic space by a polar diagram. The figure is shown in [24]. The central angle of the polar diagram represents *H*, the radius *S*, and the vertical axis *L*, which stand for hue, lightness, and saturation, respectively.

The color is a kind of signal in nature, so HLS enables the intuitive method of color science to be related to signal principal parameters [13]. *H* gives the dominant frequency component. In the circumstance of *H* is 0° represents a low frequency signal and *H* is 270° means a high-frequency signal. *L* yields effective strength of the signal (i.e., the higher cumulative energy of the signal, the larger is *L*). The range of normalized *L* is 0 to 1. *S* is the sign of the spread of contributions between the data of the signal. The range of *S* is 0 to 1, and *S =* 1 corresponds to an ideal single frequency signal, whereas *S =* 0 implies an equal amplitudes signal that does not have a dominating frequency [25].

### 3.3. Chromatic Transformations

Red, green, and blue (R, G, B) an be seen as the three non-orthogonal processors of the color signal that is able to discriminate changes in spectral distributions [12].

**Figure 3:**
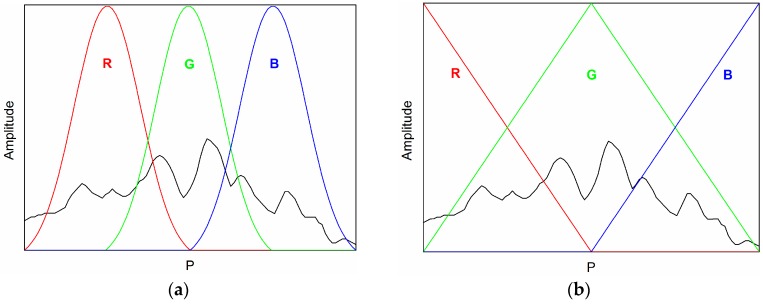
Two kinds of chromatic processor profiles: (**a**) Gaussian functions; and (**b**) triangular functions. R: red, G: green, B: blue.

Characteristics shown in Figure 3a,b are typical of the R, G, B tristimulus chromatic structure. Figure 3a shows three Gaussian functions overlapping at half-height, which are named processors. Parameter *P* can be the time or frequency for different domains. Figure 3b shows the triangular functions.

The linearity of the chromatic parameters is decided by different kinds of the processor profiles. Triangular processors provide a more uniform sensitivity throughout the frequency range but the Gaussian processors can provide regions of higher sensitivity, although they may not be so uniformly distributed. The triangular processors with half-height overlaps (as Figure 3b shows) correspond to the situation that frequency variation is linear throughout the range covered by the three processors [25]. Therefore, triangular processors as Figure 3b demonstrates are chosen in this research.

Processor responses *R*(*P*), *G*(*P*), *B*(*P*) are functions of the parameter *P*. *F*(*P*) is a signal that varies with *P* which is time or frequency.

The output of a processor *X_o_*(*P*) (*X* can be R, G or B) addressing the signal *F*(*P*) is:

(4) $$X_{o}=\int_{P}X(P)F(P)dP$$

In color science, parameters *H*, *L*, *S* are transformed from the processors’ outputs *X_o_* (*X_o_* is R, G or B). The formulas relating *X_o_*(*P*) to the *H*, *L*, *S* are [12]:

(5) $$H=\left\{ \begin{array}{ll} 240-\frac{120*g}{g+b} & r=0\\ 360-\frac{120*b}{b+r} & g=0\\ 120-\frac{120*r}{r+g} & b=0 \end{array}\right.$$

(6) L=(R+G+B)/3

(7) $$S=\frac{\max(R,G,B)-\min(R,G,B)}{\max(R,G,B)+\min(R,G,B)}$$

where:

(8) r=R−min(R,G,B)

(9) g=G−min(R,G,B)

(10) b=B−min(R,G,B)

In practice, Equations (8)–(10) mean that either *r*, *g*, or *b*, or all of them, needs to be zero. This algorithm of color science provides the principle of a convenient method for processing outputs from chromatic processors.

It can often be preferable to display *H*, *L*, and *S* as two 2-D polar diagrams rather than a 3-D polar diagram to simplify the observation of trend patterns [23].

## 4. Local Features of UHF Signal in the Chromatic Space

Figure 2b shows frequency spectra of the UHF signals acquired by the four UHF sensors. The amplitude of the signal is always greater than zero in the frequency domain. The spectra are divided into 10 sections, each has a bandwidth of 0.3 GHz. The chromatic methodology has no limitation on the length of the signal for processing, and the parameters *H*, *L*, *S*, calculated by Equations (5)–(7) represent the features in the processed scope of the signal. In this part, the signal obtained by UHF-B are processed as a representative, and each section of the spectrum is transformed into chromatic space. The local features of the UHF signal are obtained as three arrays of length 10. Similarly, the signal energy content, the average frequency and the (RMS bandwidth)^2^ are calculated according to Equations (1)–(3) in 10 sections. These three parameters are compared with the lightness, hue, and saturation which are in the chromatic space, respectively.

**Figure 4:**
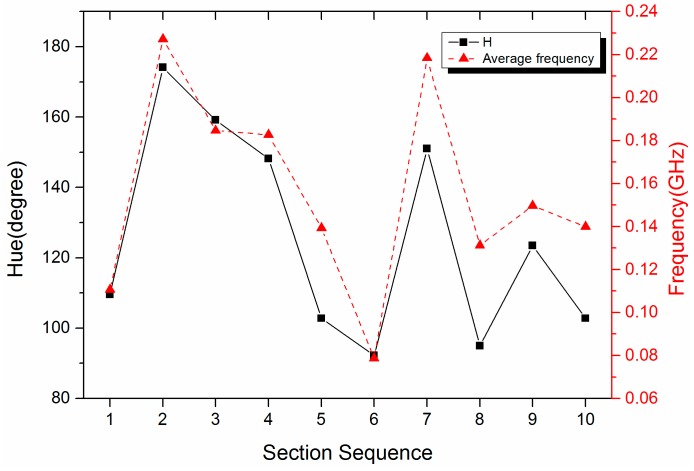
Comparison of waveforms between parameters *H* and the average frequency of the UHF-B frequency spectrum.

Figure 4 demonstrates the variation of the average frequency and the *H* value in every section. The parameter ω in Equation (2) is not the real value along the ω axis, but the range of 0–0.3 GHz for every section. Otherwise, the results of the average frequency is a curve that is an approximate linear frequency modulation signal (chirp signal) from 0 to 3 GHz, and it cannot represent local features of the spectrum in every section. Therefore, the range of the average frequency is from 0 to 0.3 GHz (the scope of Section 1).

The whole variation trend of the *H* value is basically identical to the average frequency. There is a significant rise between Sections 1 and 2. This means that the components concentrate at the low-frequency part in Section 1, i.e., the frequency intensity is on the left even more. It can be seen in the spectrum (Figure 2b) that there is a peak at 0.06 GHz. Meanwhile, several lower peaks are located on the right. Thus, the average frequency of Section 1 is about 0.11 GHz. Two high peaks are on the right side of Section 2; that is, the high-frequency part. Therefore, the average frequency of Section 2 reaches 0.23 GHz. Although there is the highest peak of the whole spectrum in Section 4, the location of it is on the right of this section. As a consequence, the average frequency of Section 4 is about 0.18 GHz instead of the highest one. It can be analyzed following a similar method for other sections. The *H* value and the average frequency represent the distribution of the frequency components in the computation interval, thus, it is independent of the intensity and energy of the signal.

**Figure 5:**
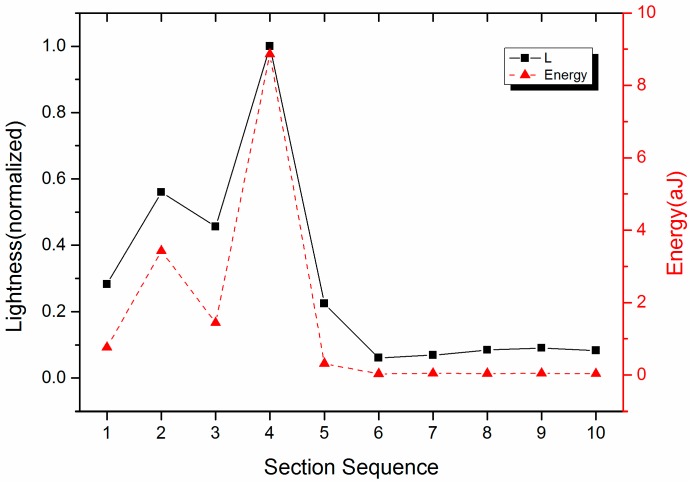
Comparison of waveforms between parameters *L* and the energy of the UHF-B frequency spectrum.

Figure 5 shows the energy and the *L* value (lightness) in each section. The energy is the integral to $F^{2}[m]$ in the section. There is the highest peak in Section 4, so the maximum energy is in this section, followed by Section 2. The intensity is very weak from Sections 6–10; as a result, the energy is 0 approximately. The variation trend of the *L* value is consistent with the energy. The *L* value is calculated by Equation (6), and it is related to the outputs of the RGB processors which are obtained by Equation (4). The *L* value has a linear relationship with the amplitude of the three processors, which are set to 1 in this paper. In order to make the *L* value in the range of [0, 1], *L* is normalized. Figure 5 shows the normalized *L* value, and the largest value of *L* is 1.

**Figure 6:**
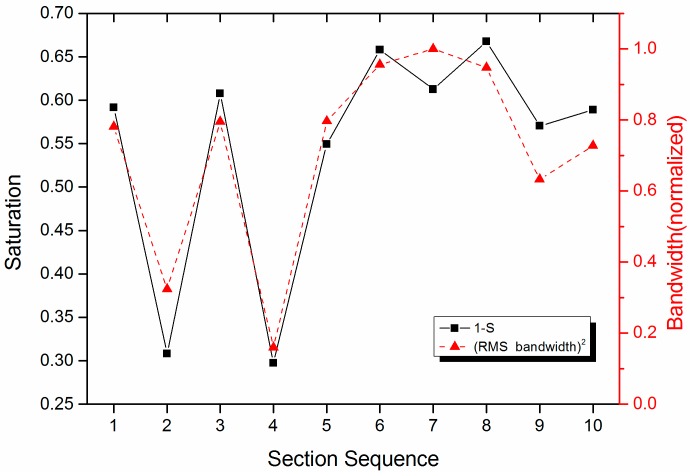
Comparison of waveforms between parameters *S* and the (RMS bandwidth)^2^ of the UHF-B frequency spectrum.

Figure 6 illustrates the variation trend of *S* value and (RMS bandwidth)^2^ in every section. *S* means *saturation* in color science, i.e., the purity of the color. For example, the saturation of red is more highly saturated than rubine. Corresponding to the physical implication, a beam of light may be made up of many of monochromatic lights with different wavelength. The more wavelength there are, the more scattered the color is, and the purity of the color is lower. On the contrary, the purity of the monochromatic light is high. The *S* value is in the range of [0, 1] according to Equation (7), and *S =* 1 corresponds to an ideal single frequency signal, whereas *S =* 0 implies an equal amplitudes signal. Then the 1 *– S* value is the opposite case exactly. Therefore, the large 1 *– S* value corresponds to the wide bandwidth in frequency domain. The variation trend is basically identical for the 1 *– S* value and the (RMS bandwidth)^2^, which agrees quite well in Figure 6. They are the lowest in Sections 2 and 4. Due to the remarkable peaks in the spectra of these two sections, the bandwidth of the signal is low.

The values *H*, *L*, and *S* obtained by the chromatic methodology have good consistency with the average frequency, the signal energy content and the (RMS bandwidth)^2^. Chromatic methodology provides a more efficient algorithm. The floating point operations (flops) are utilized to measure the efficiency of numerical algorithm [26]:

a+b*c⇒

The flops of conventional frequency parameters and chromatic parameters are listed in Table 1. The processors’ responses (Equation (4)) have most of the floating point operations in the chromatic methodology. Therefore, in order to obtain three frequency characteristics, the chromatic methodology is highly efficient and saves half of the computational cost.

In the arithmetic of chromatic processing Equations (4)–(10) can be used instead of complicated Equations (1)–(3). The parameters represent characteristics of the signal in frequency domain. The division in this paper is only a demonstration. The range of spectrum section and the processor scope can be set manually as the range which is interested in, or the region where the dominant components locate such as Sections 2 and 4. For different dimensions of the GIS tank or antenna responses, the adjustment of the division is also necessary.

## 5. Propagation Characteristics of UHF Signal Based on Chromatic Methodology

In the previous part, local features of the UHF signal processed by the chromatic methodology are investigated in every section of the frequency spectrum. If the research scope is enlarged to the whole spectrum from 0 to 3 GHz, one group of parameters *H*, *L*, and *S* can be obtained. The UHF signals acquired by four sensors can be compared for the propagation characteristics research in the chromatic space.

**Figure 7:**
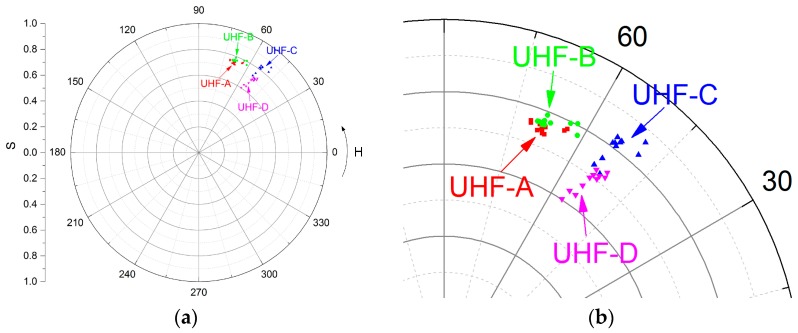
Distribution of 10 groups of UHF signals obtained by the four sensors in the *H-S* polar diagram: (**a**) global diagram; and (**b**) enlarged diagram. (UHF: ultra-high frequency).

In this part, UHF signals are acquired under the same experimental condition. The processing length is the whole spectrum. Parameters *H*, *L*, and *S* are displayed in the *H-S* polar diagram, as Figure 7a shows. Points with the same color and symbol are recorded by the same sensor at different times.

*H* values of UHF-A and UHF-B are about 70° which are larger than UHF-C and UHF-D, and it means that signals are with more high-frequency components. *H* values of UHF-C and UHF-D are about 70°, so the average frequencies are lower than UHF-A and UHF-B. Figure 2b shows that the spectra of UHF-A and UHF-B have the highest peak at 1.1 GHz, while the highest peak is at about 0.5 GHz for UHF-C and UHF-D.

There are more high-frequency components before the L-branch, and the low-frequency components enhance after passing the L-branch. The variation of the frequency intensity in different frequency bands is related to the electric field direction of the different electromagnetic wave modes and the direction of the sensor.

*S* values of UHF-B is slightly larger than UHF-A, because the peak at the same place in the spectrum reduces a very small amount from UHF-A to UHF-B. *S* values of UHF-D are remarkably less than UHF-C. The signal attenuation at UHF-D leads to the inconspicuous peaks and the more even intensity distribution in the frequency spectrum. Thus, the bandwidth increases and the *S* value decreases oppositely from UHF-C to UHF-D.

The quantitative descriptions of *H*, *L*, and *S* values are capable to be used for PD defect localization. Chromaticity changes *d_HLS_* can be quantified via the *H-S* polar diagram and *L* axis individually, i.e., chromaticity changes *d_HS_*, *d_L_* on the *H-S*, *L* plots, respectively, may be defined as follows [13]:

(11) $$d_{HS}=[{\left(S_{A}\cos H_{A}-S_{B}\cos H_{B}\right)}^{2}+{\left(S_{A}\sin H_{A}-S_{B}\sin H_{B}\right)}^{2}{]}^{1/2}$$

(12) $$d_{L}=\left|L_{A}-L_{B}\right|$$

where *H_A_*, *L_A_*, and *S_A_* are the initial values of the chromatic parameters, *H_B_*, *L_B_*, and *S_B_* are the changed values.

Figure 7b shows the significant discrimination of the UHF signals before and after the L-branch. The averaged *d_HS_* values between two sensors before and after the L-branch, respectively, are shown in Table 2. The means of the *d_HS_* values of the intervals across the L-branch is 0.2 approximately. Meanwhile, the standard deviations represent the *d_HS_* values points tend to be close to the means of the *d_HS_* values set. Therefore, the *d_HS_* value can be used to judge if UHF signals pass the L-branch.

At the straight intervals UHF-A to UHF-B and UHF-C to UHF-D, the resolving ability of *d_HS_* values are limited, as Figure 7b shows. However, *d_L_* values discriminate sensor positions remarkably. The *d_L_* values of intervals UHF-A to UHF-B and UHF-C to UHF-D shown in Table 3 are 4.79 and 4.87 (unnormalized), respectively.

The distances between the corresponding sensors are 630 mm and 820 mm. Consequently, the resolutions for the defect location are 0.00760 mm^−1^ and 0.00594 mm^−1^ before and after passing the L-branch. The resolution reduces at the interval UHF-C to UHF-D by reason of the EM wave reflection on the end of the basbar tank. The signal strength has less attenuation on the same distance.

The *H-S* points distribution in the polar diagram can distinguish UHF signals acquired by different sensors. The propagation properties of UHF signal in L-shaped GIS is revealed based on chromatic methodology. It also provides an insight into the signal discriminating capabilities of this method. The defect location can be estimated by the combination of *d_HS_* and *d_L_* values effectively.

## 6. PD Defect Pattern Recognition

**Figure 8:**
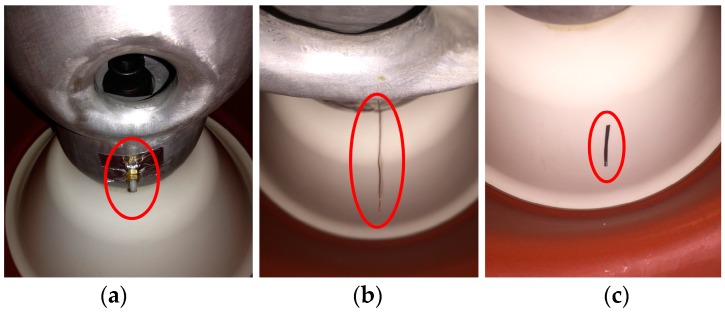
Typical PD defect patterns in actual 252 kV GIS tank: (**a**) floating electrode; (**b**) metal protrusion; and (**c**) particles on the spacer surface.

One of the main goals of UHF signal processing and feature extraction is to recognize different PD defect patterns in GIS. There have been numerous research works about this aspect in the past decade. Chang developed a classifier which derives features directly from the original waveform in time-domain [27]. Li et al. improved the differential box-counting method and used fractal features and statistical parameters of PRPD grey images to recognize typical PD patterns [28]. In this paper, new features derived from the chromatic space are proposed and used for PD defect recognition. Each UHF signal is represented by a group of 3-D features which have low dimension. Three typical PD defect patterns are mounted in the actual GIS tank, floating electrode, metal protrusion, and particle on the spacer surface, as Figure 8 shows.

One-hundred groups of UHF signals are recorded in each pattern and the applied voltage is the same. Only signals of UHF-A are processed to classify different patterns. As a pattern recognition algorithm, the support vector machine (SVM) is a popular machine learning method for classification [29], which is applied in this work.

The kernel function maps the feature vector into the higher dimensional space for the purpose of finding a linear separator in the new space [30]. The Gaussian radial basis function (Gaussian-RBF) kernel is selected in this work. The Gaussian-RBF is defined as:

(13) $$K(x_{i},x_{j})=\exp(-\gamma {\left\|x_{i}-x_{j}\right\|}^{2})$$

where γ is the kernel parameter controlling the flexibility of classifiers [30]. In the SVM algorithm, *C* is a cost penalty parameter for misclassifications. In order to obtain the best classification results, parameters *C* and γ are optimized using the five-fold cross-validation in this model.

**Figure 9:**
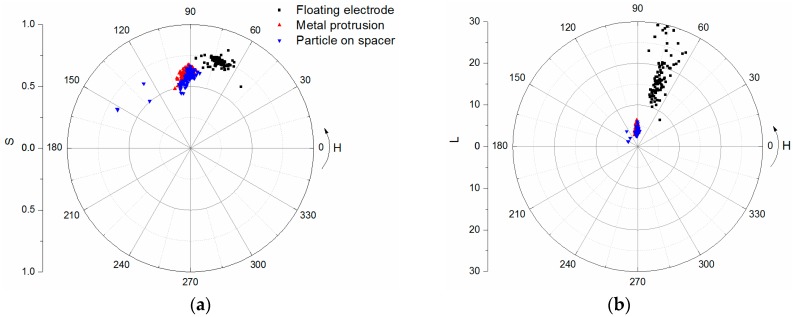
Chromatic parameters distribution of different PD patterns in polar diagram: (**a**) H-S; and (**b**) H-L.

Chromatic parameters *H*, *L*, and *S* are extracted from each UHF signal; half of the groups of data are the training set, and the others are the test set. The distribution of chromatic parameters in polar diagrams are shown in Figure 9. The UHF signal of the floating electrode has more low-frequency components than other two defects. There are some points of metal protrusion and particles on the spacer surface overlapping in the polar diagrams. However, they can be classified well in the 3-D chromatic space. At the final stage, the classification results of the SVM model based on the chromatic parameters *H*, *L*, and *S* are listed in Table 4, and the whole classification accuracy is 86.67%.

**Figure 10:**
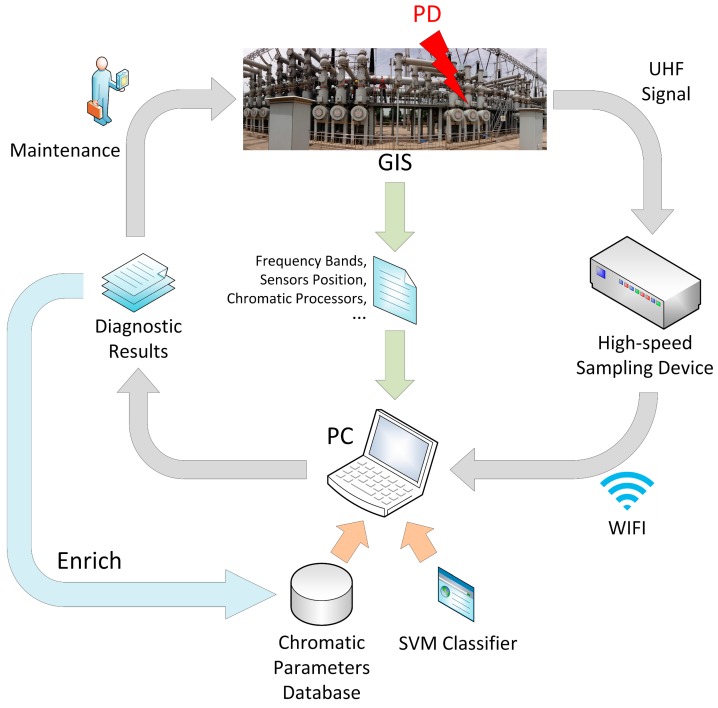
Flow chart of PD pattern recognition using chromatic methodology (GIS: gas-insulated switchgear; PD: partial discharge; UHF: ultra-high frequency; WIFI: wireless fidelity; SVM: support vector machine; PC: personal computer).

Laboratory experiments indicate that the chromatic methodology has good performance and great potential for use with field PD detection. Figure 10 summarizes the procedure of PD pattern recognition using chromatic methodology.

First, input the GIS structure-related parameters to the PC, such as frequency bands of specific EM-wave modes, the positions of UHF sensors, and the profile and width of chromatic processors.

Then, the high-speed sampling device acquires UHF signals once PD occurs inside GIS. The device transmits UHF signal waveform to a portable PC through wireless communication. The relationship between the software system and sampling devices is one-to-many, which is convenient and efficient in field applications. All of the data post-processing and pattern recognition are done on a computer.

Next, the trained SVM classifier runs on a computer. The SVM model classifies the PD defect pattern based upon the chromatic parameters database. The chromatic parameters database is obtained by a large amount of experimental results before commissioning.

Finally, the technical staff can construct the maintenance plan with the assistance of the diagnostic results. On the other hand, the results of maintenance enrich the database. The SVM classifier will be more and more powerful to recognize different PD defects. When using different UHF sensors, the detection system also applies to other high voltage equipment, such as transformers, power cables, and rotating machines.

## 7. Conclusions

This paper investigates the UHF signal processing of PD in GIS using the chromatic methodology, which is novel in PD detection. The principle and application of chromatic methodologies are introduced. The UHF signals acquired by the PD experiments are transformed to the chromatic space, while the features in the local and global scopes are researched. Finally, chromatic parameters are utilized to classify typical PD patterns and good performance is obtained. The conclusions are summarized as follows:

- Chromatic monitoring creates an analogy with the signal and color, and the parameters in chromatic space are related to the color features. Hue, lightness, and saturation represent the dominating frequency, signal strength, and effective signal bandwidth of the color, respectively.
- The frequency spectrum is divided into 10 sections, and the local features in the chromatic space are investigated. The parameters *H*, *L*, and *S*, which can represent the features have a good consistency with the average frequency, the signal energy content, and the (RMS bandwidth)^2^ of the signal in frequency domain.
- The scope of the chromatic processing length can be set manually as the range of interest, or the region where the dominant components locate. If the research subject is the whole spectrum, the chromatic parameters can be used to distinguish UHF signals acquired by different sensors. The *d_HS_*, *d_L_* parameters of different UHF sensors in the chromatic space have an effect for the PD source localization. It reveals the propagation characteristics of UHF signals in the L-shaped GIS.
- The chromatic processing is powerful in signal discrimination, and makes an efficient, sparse representation of the UHF signal. Three typical PD defect patterns are classified accurately. The results of PD defect pattern recognition verify and prove the usefulness and feasibility of the chromatic methodology. The detection system and the procedure of PD pattern recognition using chromatic methodology is summarized.

## Figures and Tables

**Table 1:** Floating point operations of different parameters.

Parameters ^1^	*E_f_*	*ω_c_*	*B*^2^	R	G	B
Flops ^2^	*M*	*2M*	*3M*	*M*	*M*	*M*
Flops in Total	*6M*			*3M*		

^1^ Defined in Equations (1)–(4); ^2^ *M* is the length of the discrete spectrum.

**Table 2:** *d_HS_* values of the intervals across the L-branch.

Intervals	*d_HS_*	Standard Deviation (σ)
UHF-A to UHF-C	0.2154	0.0064
UHF-A to UHF-D	0.2011	0.0182
UHF-B to UHF-C	0.1988	0.0296
UHF-B to UHF-D	0.2014	0.0204

**Table 3:** *d_HS_* values of the intervals across the L-branch.

Intervals	*d_L_*	Distance (mm)	Resolution (mm^−^^1^)
UHF-A to UHF-B	4.79	630	0.00760
UHF-C to UHF-D	4.87	820	0.00594

**Table 4:** Classification results of the SVM model in the chromatic space.

PD Defect Pattern	Number of Recognition	Recognition Rates
Floating electrode	50	100%
Metal protrusion	44	88%
Particle on the spacer surface	36	72%
**Total**	**130**	**86.67%**
